# Adverse Effects of Ocean Acidification on Early Development of Squid (*Doryteuthis pealeii*)

**DOI:** 10.1371/journal.pone.0063714

**Published:** 2013-05-31

**Authors:** Maxwell B. Kaplan, T. Aran Mooney, Daniel C. McCorkle, Anne L. Cohen

**Affiliations:** 1 School of Biology, Medical and Biological Sciences Building, University of St Andrews, St Andrews, Fife, United Kingdom; 2 Biology Department, Woods Hole Oceanographic Institution, Woods Hole, Massachusetts, United States of America; 3 Geology and Geophysics Department, Woods Hole Oceanographic Institution, Woods Hole, Massachusetts, United States of America; National Institute of Water & Atmospheric Research, New Zealand

## Abstract

Anthropogenic carbon dioxide (CO_2_) is being absorbed into the ocean, altering seawater chemistry, with potentially negative impacts on a wide range of marine organisms. The early life stages of invertebrates with internal and external aragonite structures may be particularly vulnerable to this ocean acidification. Impacts to cephalopods, which form aragonite cuttlebones and statoliths, are of concern because of the central role they play in many ocean ecosystems and because of their importance to global fisheries. Atlantic longfin squid (*Doryteuthis pealeii*), an ecologically and economically valuable taxon, were reared from eggs to hatchlings (paralarvae) under ambient and elevated CO_2_ concentrations in replicated experimental trials. Animals raised under elevated *p*CO_2_ demonstrated significant developmental changes including increased time to hatching and shorter mantle lengths, although differences were small. Aragonite statoliths, critical for balance and detecting movement, had significantly reduced surface area and were abnormally shaped with increased porosity and altered crystal structure in elevated *p*CO_2_-reared paralarvae. These developmental and physiological effects could alter squid paralarvae behavior and survival in the wild, directly and indirectly impacting marine food webs and commercial fisheries.

## Introduction

Global atmospheric carbon dioxide concentrations have increased significantly from pre-industrial levels of c.280 ppmv to approximately 390 ppmv today (Earth Science Research Laboratory Global Monitoring Division 2012) as a result of human activity [1]. The world's oceans are a carbon sink and have absorbed nearly a third of all anthropogenic CO_2_ since 1800, without which atmospheric levels would be much higher than they are now [2]. To date, changes in marine carbon chemistry as a result of CO_2_ uptake include reductions in seawater pH, carbonate ion concentration, and aragonite (CaCO_3_) saturation state [3]. Projections under the Intergovernmental Panel on Climate Change (IPCC) A1F1 scenario suggest that atmospheric CO_2_ will increase to ∼1000 ppmv by the year 2100 [4], [5], with further increases up to 2000 ppmv by the year 2300 [1]. These increasing atmospheric concentrations will lead to further reductions in the global average surface-water pH of 0.3–0.4 units by the year 2100 [6]. Coastal and high latitude pH levels may be decrease more and reductions will likely occur sooner [1], [7].

A number of studies have examined possible organismal responses to a more acidic ocean. Some calcifying taxa have reduced calcification rates under acidic conditions [6] including corals (reviewed in [8]), foraminifera (e.g. [9]), coccolithiphores (e.g. [10]), bivalves (e.g. [11], [12]), and pteropods (e.g. [13]). However, some other species demonstrate increased calcification rates, including echinoderms [14]–[16], decapod crustaceans [15], teleost fish [17], and some juvenile cephalopods [18], [19].

Impacts on development and early life history are a particular concern [20]–[25] because young animals may not be as resilient to physiological stress as juveniles or adults [21], [26]. Atlantic cod (*Gadus morhua*) larvae reared under ocean acidification conditions (1800 and 4200 μatm) exhibited severe to lethal tissue damage in multiple organs; effects were more pronounced with increasing *p*CO_2_ [26]. In addition, early exposure of estuarine fish larvae (*Menidia beryllina*) to elevated *p*CO_2_ (up to ∼1000 μatm) reduced survival and larval length [21]. These effects raise concern as recruitment cohorts lay the foundation for population success.

Squid play a major role in many marine ecosystems. They are both predators and prey for a variety of taxa across multiple trophic levels [27]. As a prey resource, squid support many global finfish fisheries including various tuna, billfish and groundfish species [28]–[30], and several squid species play an important role in linking apex predators with the squids' mesopelagic prey [31]–[33]. For example, breeding success of the Grey-headed albatross (*Thalassarche chrysostoma*) is significantly and positively correlated with the abundance of the squid *Martialia hyadesi* in their diet [34].

Many squid species constitute or support productive fisheries, and estimated global squid catches are near 3 million t yr^−1^ [28]. In the United States, the market squid (*Doryteuthis opalescens*) was California's largest fishery in 2009 and 2010 by weight and value [35]. However, squid can be affected by changes in surrounding environmental conditions [36], [37]. For example, the population size of *Illex argentinus* in the Southern Ocean has been linked to interannual variation in sea-surface temperature (SST) in a likely hatching area, with SST of 16–18°C most favorable for successful recruitment [38].

A limited number of studies have investigated cephalopod responses to pH or *p*CO_2_. Work has focused on the cuttlefish and experiments suggest that pH is an important measure of water quality for certain squid species [39]. Elevated *p*CO_2_ impairs cephalopod oxygen transport capabilities because oxygen binding to the cephalopod respiratory protein haemocyanin is highly pH dependent [40]. In the European cuttlefish (*Sepia officinalis*), dorsal mantle lengths and body mass of embryos and hatchlings were significantly shorter under elevated *p*CO_2_ (∼3700 μatm) compared to control concentrations [41]. It was suggested the extreme hypercapnia (elevated blood *p*CO_2_) could induce metabolic depression in developing hatchlings because the degree of sensitivity to reduced seawater pH may be linked to the presence and capacity of ion regulatory structures, which may not be fully formed during early cephalopod life stages [41]. Despite these effects, another experiment that exposed hatched *S. officinalis* to very high CO_2_ concentrations (∼4000 and ∼6000 μatm) over a 6-week period found no difference in mantle length between treatments [18]. These seemingly disparate findings between experiments may result from the different life stage at which measurements were taken (i.e., juvenile cuttlefish may have better developed acid-base regulatory abilities than developing embryos).

In addition to physiological impacts, internal calcified structures such as squid statoliths may also be affected by reduced seawater pH [18], [19], [42], [43]. Located in paired statocysts and analogous in function and similar in structure to fish otoliths, statoliths are vital for sensing gravity and movement [44]. Squid statolith formation is initiated directly after gastrulation during early embryonic development [45]. Initial results for one squid species, *L. vulgaris*, suggest statolith surface area may increase with greater *p*CO_2_ levels (850 and 1500 μatm) [42]; however, aragonite saturation states were not calculated, which makes it difficult to assess these changes. Even small impacts to statolith growth are of concern because improper formation can severely impact balance and orientation [46].

Elevated *p*CO_2_ (∼6000 μatm) resulted in increased calcification rates of the cuttlebone in adult *S. officinalis*. A more recent experiment on cuttlefish embryos and juveniles also suggests that cuttlebone calcification rates increase even under more moderate levels of seawater *p*CO_2_ (∼800 and ∼1400 μatm) [19]. As these studies vary in species, life stage and *p*CO_2_ levels, it is still unclear how calcification in cephalopods is affected by *p*CO_2_.

In particular, there is a need for a focused study to examine how ocean acidification may impact squid. Here we investigate the impacts of elevated *p*CO_2_ on the early life history of Atlantic longfin squid *Doryteuthis pealeii* (formerly *Loligo pealeii*) paralarval development (time to hatching and size at hatching), statolith size, and statolith morphology. The study species, *D. pealeii*, is of significant commercial importance in the North Atlantic [47] and is considered a key prey item for a variety of marine mammal, seabird, and finfish species [27], [31].

## Materials and Methods

Experiments were conducted at the Woods Hole Oceanographic Institution between June-August 2011. The use and care of the animals was performed with approval from the Woods Hole Oceanographic Institution's Animal Care and Utilization Committee (IACUC). All necessary permits were obtained for the described studies including the collection of adult squid. These were gathered by the Marine Biological Laboratory, which has a research permit issued by the Massachusetts Division of Marine Fisheries to collect invertebrates in various life stages for research and education (permit number 152087). No specific permissions were required for these locations/activities because they fall under the permit provisions. The collecting location was not privately owned or protected in any way and the field studies did not involve endangered or protected species.

### Collection and husbandry

Squid were captured by trawl in Vineyard Sound on two occasions in 10–30 meters of water. Temperature in the area at the time of capture was 16.7°C and 17.9°C, and salinity was 30.7 and 30.8, respectively (data from the 12 meter node at the Martha's Vineyard Coastal Observatory). These values are typical for longfin squid recruits, which are primarily found in water ranging from 6–35 m depth, 4–28°C, and with salinity of 30–37 between the spring and fall [29]. Similar to some other inshore squid species, *D. pealeii* is able to tolerate temperature and salinity variations [39]. Adults in healthy condition (free of cuts and scrapes) were hand-selected from the group, gently placed in individual buckets, and transported from the Marine Biological Laboratory to a holding tank in the Environmental Systems Laboratory (Woods Hole Oceanographic Institution, Woods Hole, MA) within 1 hour of being caught. To encourage mating, more females than males were selected from the trawl (f:m ratio; 6∶2 and 16∶3). The holding tank (120 cm diameter; 70 cm depth) contained a layer of fine-grained sand at the bottom (∼2 cm thick). The sand was collected from a nearby beach and was rinsed thoroughly with sand-filtered seawater prior to being added to the holding tank. The holding tank was set up in a flow-through system with sand-filtered seawater that was temperature-controlled to ∼20°C using aquarium heaters and chillers. In accordance with our animal care protocols, squid were fed twice daily with live *Fundulus heteroclitus*, which were gathered from a local bay.

### Experimental set-up

Individual aquaria (1-litre Solo PET food service containers) were set up in a flow-through system at the beginning of each trial and were equilibrated with different CO_2_ concentrations. The PET containers (16 in total) were placed in a water bath in which temperature was maintained at ∼20°C (monitored using an Onset data logger (pendant model UA-002-64), which recorded ambient light intensity and water temperature every 15 minutes). The containers were covered tightly with lids in which a single hole had been cut (0.5 cm diameter), which allowed the water and gas tubing to fit snugly inside. Vineyard Sound seawater, temperature-controlled to 20°C and 5 µm-filtered, was fed into a header tank from which it flowed through 2 ‘H’-shaped equilibration chambers. Seawater in chamber 1 was continuously equilibrated with air pumped from an indoor air compressor, while seawater in chamber 2 was equilibrated with the air from the same source enriched with CO_2_ using Aalborg Mass Flow Controllers (model GFC17 and GFC37). Both gas mixtures passed through air stones in the equilibration chambers. The concentrations of CO_2_ in the gases bubbled in the two sets of aquaria were set to 390 μatm (control) and 2200 μatm (treatment), targeting pH levels of 8.0 and 7.3 for the control and elevated *p*CO_2_ levels respectively. CO_2_ concentrations of the gases were analyzed weekly using a Qubit Systems CO_2_ Analyzer (model s151) with reference to 3 known commercially prepared standards (1036, 362 and 0 ppm). Gas concentrations in both treatments remained stable for the duration of the experiment (mean ± SE; control: 394±6 ppm; treatment: 2267±10 ppm.

Water from the equilibration chambers entered a PVC manifold from which it was supplied individually to the containers to ensure that the egg capsules were well oxygenated and in order for metabolic byproducts to be expediently removed from the containers (as in [48], [49]). Flow to each container was approximately 21 liters d^−1^. Each container was also bubbled individually with the same air or air + CO_2_ mixture to ensure continued equilibration. Bubbling rates and water flow rates in the “H”-shaped equilibration columns (upstream), and in the experimental cups (downstream) were adjusted so that pH was on a plateau and not sensitive to small fluctuations in water flow, water chemistry, or gas flow. Outgoing water dripped out of the container through a hole in the side of the container covered in 500 µm mesh to prevent loss of larvae. Water was circulated through the system for several days prior to introducing the eggs, during which the pH in each treatment was tested every other day using a pH meter (Orion 3 Star Plus model 1212001, ThermoElectron Corporation) to ensure that target pH levels had been reached and remained stable. The experiment was carried out in a windowless room that was maintained on a 12∶12 light:dark photoperiod by 4 ceiling-mounted fluorescent bulbs.

Multiple females laid several egg capsules in one large egg cluster after 1–2 days in the holding tank prior to the start of both experimental trials. Each egg capsule may contain between 100–200 fertilized eggs, with considerable inter-capsule variability in the number of eggs per capsule both within and between females [50]. Furthermore, female *D. pealeii* are able to store sperm from multiple males [51], and their egg capsules are known to contain eggs fertilized by multiple males [52]. Thus, it was likely these egg capsules contained fertilized embryos from multiple males.

The morning after eggs were laid, 2 randomly selected egg capsules from the egg cluster were added to each of 6 containers per trial (3 containers per CO_2_ concentration per trial). This balanced the need for numerous embryos with the feasibility of measuring paralarvae immediately after hatching. An additional 4 containers were included in the water bath as blanks (2 per CO_2_ concentration) and contained no eggs throughout the experiment for comparative purposes so that seawater chemistry measurements could be taken independent of potential biotic effects on chemistry parameters.

### Seawater Chemistry

Seawater pH was measured using a pH meter every other day and by spectrophotometer weekly throughout the experiment in a method adapted from Clayton & Byrne [53] and Dickson *et al.* [54]. Samples for spectrophotometric pH analysis were first taken on 6 July 2011 (day 5 of trial 1) and were taken weekly thereafter from a subset of aquaria. Electrode-based pH measurements were converted from the NBS scale to the total scale and were used for monitoring purposes only; spectrophotometric pH measurements, also expressed on the total scale, were used for seawater chemistry calculations.

Salinity samples were collected in 120 mL glass bottles weekly concurrently with the samples for spectrophotometric analysis, but were analyzed at a later date. Total alkalinity (A_T_) samples were also taken weekly in plastic acid-washed 20 mL scintillation vials and poisoned with 11 µl of saturated mercuric chloride (HgCl_2_). These samples were analyzed using automated Gran titrations of 1 mL samples, run in duplicate and standardized using certified reference materials (from the laboratory of Andrew Dickson, SCRIPPS Institution of Oceanography) (method adapted from Holcomb *et al*. [49]). If there was a discrepancy of more than 4 µequiv/kg, duplicate samples were run again. Using CO2sys software, temperature, pH, salinity, and A_T_ values were used to calculate aragonite saturation state values (Ω_Arag_) in each treatment [55], [56] using dissociation constants from Mehrbach *et al.* [57] refit by Dickson & Millero [58], sulphate constants from Dickson [59], and carbonate mineral solubilities from Mucci [60].

### Measurement Protocol

Containers were checked for hatching daily, and time to first hatching was recorded. Containers continued to be inspected every 24 hours until all eggs had hatched (18 days in trial 1; 19 days in trial 2 from day of egg laying to last hatching). On the day they were observed, all hatchlings were removed from their container and counted in order to calculate the number of animals hatched per day. Larvae were then placed in new containers (one container per treatment containing all of the hatched larvae from that day) to separate the eggs and new hatchlings. Thus, cup densities varied. Paralarvae were not fed, because they hatch with a yolk sac, which fuels their initial (post-hatching) metabolic requirements [61]. Animals were not reared beyond this yolk stage because of the high mortality rate of squid raised in captivity [39].

A subset of the hatchlings (10 paralarvae d^−1^) from each of the 3 containers per treatment and trial (i.e., n = 30 animals per treatment per trial per day) was subjected to morphological analyses. Randomly selected individuals were gently lifted via pipette and placed lengthwise and dorsal-side up on a glass slide in a small drop of water. Multiple photographs of each individual were taken using a Dino-Lite Pro2 AD-413TL USB-microscope (calibrated twice daily prior to taking the first measurement of each treatment by placing a standard in the field of view) and the DinoXcope software. Only photographs of the dorsal side, of undamaged individuals, and of animals that were not moving when the image was captured, were retained for mantle length analysis. In order to facilitate rapid assessments of the hatchings, dorsal mantle length (ML) was determined from the image later using the measurement tools in the DinoXcope software.

Every day another random subset of paralarvae (n = 10 per treatment, pooled across containers) were fixed in 97% ethanol. Statoliths were dissected out of a further subset of these ethanol-fixed paralarvae (pooled across days but separated by CO_2_ treatment). Only one statolith per dissected individual was retained in order to maintain sample independence. All statoliths were soaked briefly (∼15 minutes) in a dilute bleach solution in order to dissolve any remaining tissue. This is a standard method to remove tissue from otoliths and coral skeletons before detailed mass and visual morphological measurements [62]–[64]; visual assessments of the statoliths indicated that this method did not impact morphology. We obtained 36 statoliths from control and 22 from treatment CO_2_-reared individuals available for comparison; unequal numbers resulted from the difficult nature of statolith extraction and a limited number of preserved larvae from which samples could be taken. The statoliths were mounted on stubs in a uniform orientation (anterior view) for scanning electron microscopy (SEM) using a fine paintbrush and were sputter-coated with platinum or gold. Images were collected using both a Zeiss NTS Supra 40VP with a field emissions source for the electrons and a JEOL JSM 35CF. Surface area measures were made using the programs Axiovision (Carl Zeiss, USA) and SemAfore (JEOL, Germany) with outline and ROI tools, which captured 2-dimensional surface area. These tools were calibrated individually for each SEM image using the scale bar present.

**Figure 1:**
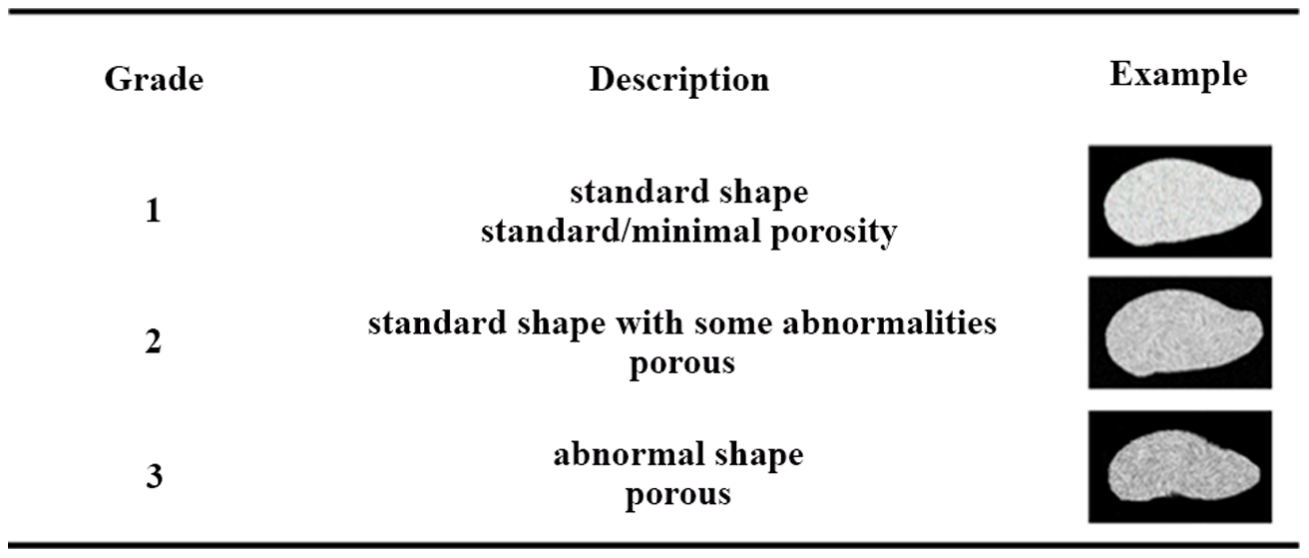
Grading criteria for statoliths according to shape and porosity. Grades were given to statoliths based on SEM images. Example diagrams show increasing porosity and malformations with increasing grade.

Statoliths were graded according to their porosity (e.g., [65]) and shape from the SEM images using a categorical grading system (Fig. 1). This method was based on a system of categorizing morphological abnormalities in developing squid by Rosa *et al*. [66] and was defined as: (1) standard statolith shape and normal/minimal porosity, (2) standard shape with some abnormalities in the surface structure, slightly porous, and (3) porous and/or abnormal shape. Standard statolith shape is well described elsewhere [44], [67]–[69]. Briefly, statoliths are oriented in the long axis approximately in line with the dorso-ventral plane of the animal [69]. Adult squid statoliths are composed of four parts: dorsal dome, lateral dome, rostrum wing, with variation between species [69]; however, paralarval statoliths are typically droplet-shaped [68]. Abnormalities were defined by varying degrees of pitting in the statolith surface and morphological deformations (i.e., deviations from the droplet shape).

**Figure 2:**
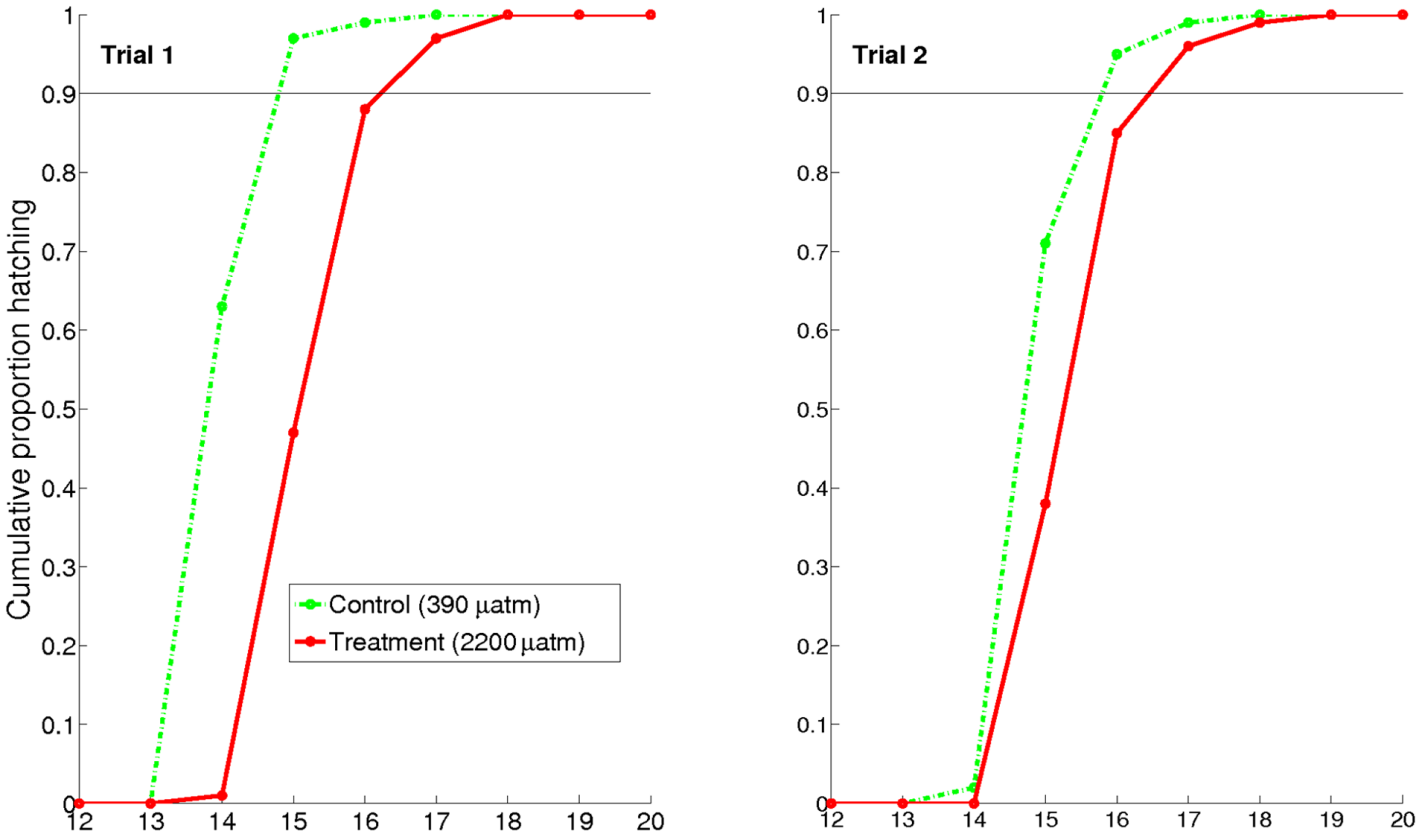
Hatching timeline for squid paralarvae over replicated trials. Paralarvae reared under control CO_2_ concentrations initiated hatching earlier. Hatching numbers were pooled across individual aquaria and the proportion hatching per day was calculated. Distributions of hatching numbers were significantly different (Chi-squared, trial 1: χ^2^ = 789.l, df  = 4, p<0.001; trial 2: χ^2^ = 197.0, df  = 5, p<0.001). Because egg capsules contained variable numbers of embryos, this figure shows the proportion hatching per day to better reflect relative differences between treatments and trials. In trial 1, hatching of control paralarvae was 90% complete (line) 24 hours earlier than of paralarvae raised under treatment CO_2_ conditions. Delays were smaller in trial 2 but still notable. Total number of paralarvae hatching (control, treatment): trial 1–577, 855; trial 2–887, 533.

Statistical analysis was carried out in PASW 18.0 and SPSS 19.0 for Windows (IBM Corporation, NY, USA). Values reported are means ± SE unless otherwise noted. One-way ANOVAs, General Linear Models (GLMs) and Chi-squared tests were used to compare between treatments and trials. Where ANOVAs were used, data were normally distributed. The hatching data represent counts in a J-by-K table where J is the number of treatments and K is the number of days. We tested the null hypothesis that the distribution of hatching across the days was the same for all treatments using a Chi-squared test. Because egg capsules contained variable numbers of embryos, Fig. 2 shows the proportion hatching per day to better reflect relative differences between treatments and trials. All results were adjusted using the Bonferroni correction, which reduces the critical p value based on the number of parameters tested.

## Results

Gas concentrations in both treatments remained stable for the duration of the experiment, and resulted in pH (total scale) of 7.86 (control) and 7.31 (treatment), and corresponding Ω_Arag_ values of 1.71 and 0.53 respectively (Table 1). Thus, the control *p*CO_2_ condition was supersaturated with respect to aragonite while the elevated *p*CO_2_ condition was under-saturated. The pH and saturation state of the control are lower than what would be expected from equilibration with air, which may reflect variation in near-shore conditions during the course of the experiment and/or incomplete equilibration of the incoming flow-through water with the control bubbled air. As such, calculated *p*CO_2_ levels (from CO2sys) were 626 μatm for the control and 2440 μatm for the treatment (averaged across trials). There were clear differences in pH between control- and treatment-*p*CO_2_-equilibrated aquaria. Temperature, salinity and alkalinity remained steady for the duration of the experiment (Table 1).

**Table 1:** Carbon chemistry measurements.

Condition	Temperature (°C)	pH_total_	Salinity	A_T_ (mmol/kgSW)	Ω_Arag_
390 µatm Blank	20.58 (0.01)	7.87 (0.02)	30.865 (0.045)	2069.7 (4.7)	1.76 (0.06)
390 µatm Trial 1	20.53 (0.01)	7.85 (0.01)	30.851 (0.046)	2067.0 (10.0)	1.68 (0.01)
390 µatm Trial 2	20.62 (0.01)	7.86 (0.01)	30.799 (0.027)	2061.1 (3.5)	1.69 (0.02)
2200 µatm Blank	20.58 (0.01)	7.32 (0.01)	30.799 (0.032)	2060.7 (5.7)	0.54 (0.01)
2200 µatm Trial 1	20.53 (0.01)	7.30 (0.01)	30.900 (0.026)	2081.3 (2.3)	0.52 (0.01)
2200 µatm Trial 2	20.62 (0.01)	7.31 (0.01)	30.756 (0.013)	2058.1 (3.9)	0.53 (0.01)

Means (SE) for seawater carbon chemistry parameters pooled across containers and trials. Ω_Arag_ calculated using CO2sys software [55], [56].

While paralarvae first hatched on day 14 of both trials, animals raised in control conditions hatched in higher proportions. On day 14 (first day of hatching) of trial 1, control animals were 62.6% hatched, whereas only 0.7% hatched under treatment conditions (Fig. 2). Hatching proportion difference on the first day of trial 2 hatching was smaller (day 14: 1.8% control; 0.0% treatment). However, the following day, control cups hatched in substantially higher proportions (70.5% control; 37.5% treatment). The time to reach 90% of total hatching was ∼24 hours greater for trial 1 paralarvae reared under elevated *p*CO_2_ than for trial 1 control paralarvae. A similar delay was apparent but shorter (<24 h) in trial 2. The temperature range from one day prior to hatching until hatching was complete was 19.9–21.2°C for trial 1 and 19.9–21.3°C for trial 2, and there were no temperature spikes that may have stimulated early hatching. The overall distributions of proportion hatching differed significantly between treatments in both trials (Chi-squared, trial 1: χ^2^ = 789.l, df  = 4, p<0.001; trial 2: χ^2^ = 197.0, df  = 5, p<0.001). Hatching was complete after 5 days in trial 1 and after 6 days in trial 2 (resulting in variable degrees of freedom between trials). Thus both initial and final (total) hatching of the CO_2_ treated embryos was delayed by approximately 24 hrs in trial 1, and by a smaller margin in trial 2.

**Figure 3:**
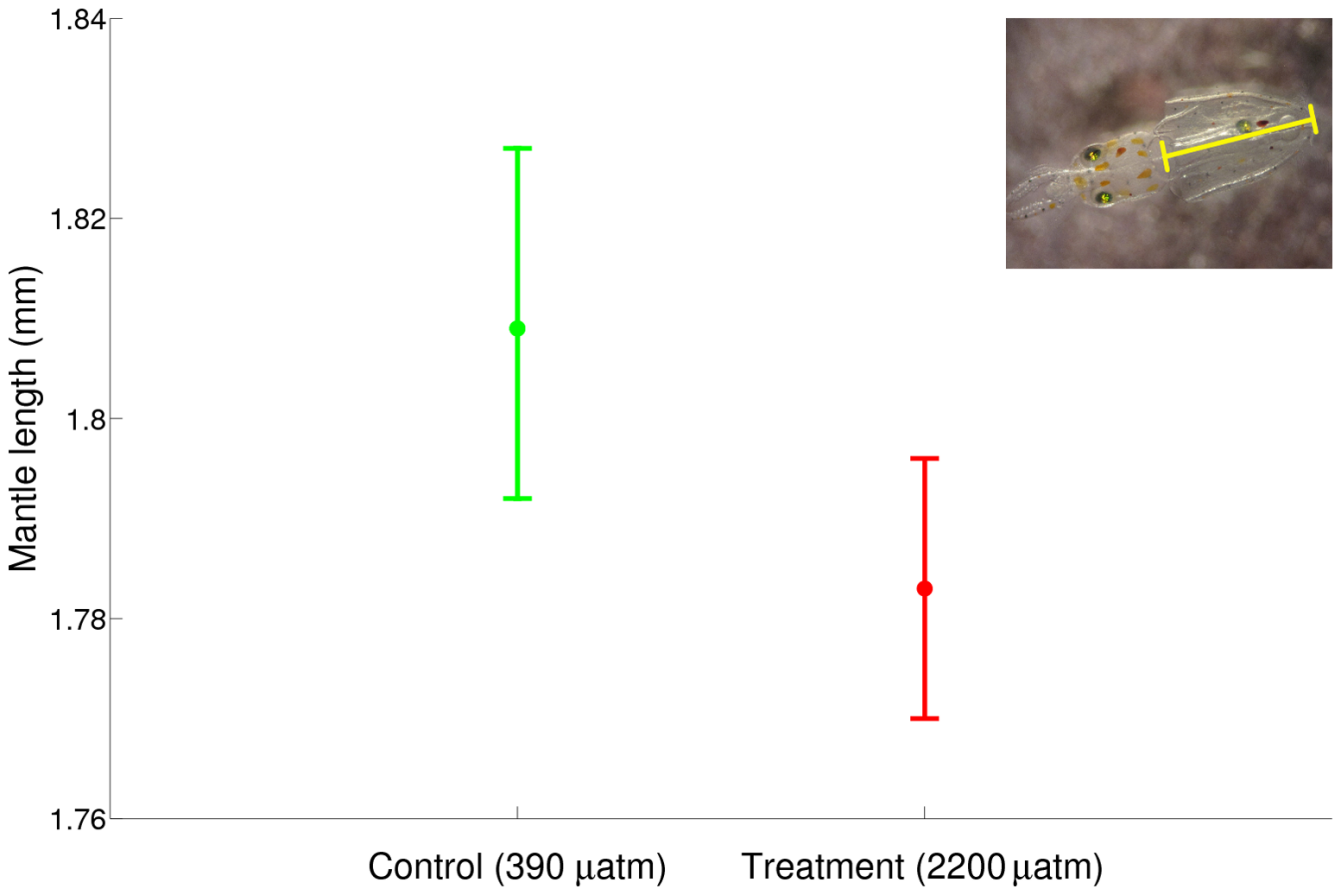
95% confidence intervals for paralarval mantle lengths of squid raised in control and treatment CO_2_ conditions. Mantle lengths were significantly longer (F_1,292_ = 9.241, p<0.003) for paralarvae reared under control conditions (1.81±0.01; n = 120) compared to treatment (1.78±0.01; n = 175).

Mantle length differed significantly between treatments (GLM with mantle length as the dependent variable and trial and treatment as factors: F_1,292_ = 9.241, p<0.003), but the differences were small. Animals raised in elevated *p*CO_2_ conditions had shorter mantles in both trials (treatment: 1.78±0.01 mm; n = 175, c.f. control: 1.81±0.01; n = 120). Differences were significant when trials were pooled (Fig. 3), and within trial 1. Trial 2 showed clear, but not significant, differences. The difference between trials in hatchling mantle lengths was significant (F_1,292_ = 13.027, p<0.001).

**Figure 4:**
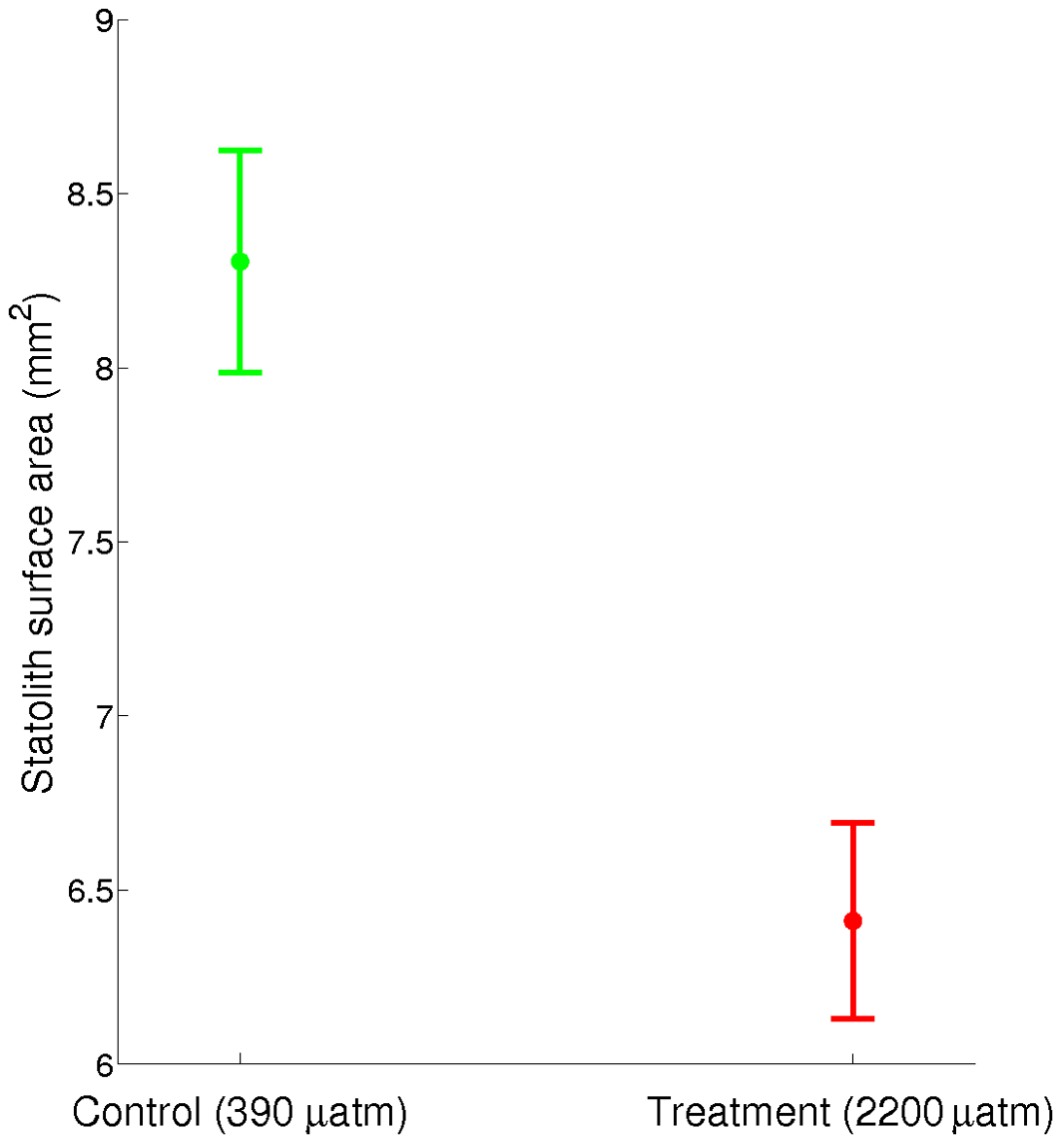
Statolith surface area. 95% confidence intervals for statolith surface area pooled across trials. Statoliths from paralarvae reared under control conditions were significantly larger in area (F_1,55_ = 70.722, p<0.001, 8.3±0.16 mm^2^) than those from animals reared in treatment CO_2_ (6.4±0.14 mm^2^).

**Figure 5:**
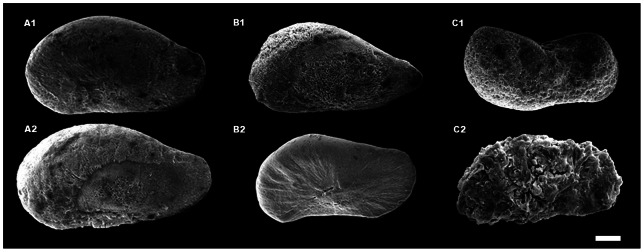
SEM images of paralarval statoliths. Six squid statoliths imaged using SEM (scale bar (white line)  = 20 μm). Statoliths of squid raised in elevated *p*CO_2_ (2200 μatm) concentrations were more porous and more irregular in shape, compared to statoliths from individuals reared under control (390 μatm) CO_2_ levels. Statoliths A1 and A2 (both control) are grade 1 and are indicative of normal paralarval statolith shape and structure. Statoliths B1 (control) and B2 (treatment) are grade 2. B1 is indicative of a moderate degree of porosity and B2 shows some malformation in structure. Statoliths C1 and C2 (both treatment) are grade 3 and both demonstrate a major degree of porosity and malformation. (A1: 138 µm long, 8.3 mm^2^; B1: 130 μm long, 7.2 mm^2^; C1: 118 µm long, 6.4 mm^2^. A2: 150 μm long, 9.9 mm^2^; B2: 122 μm long, 6.4 mm^2^; C2: 123 μm long, 6.3 mm^2^).

**Figure 6:**
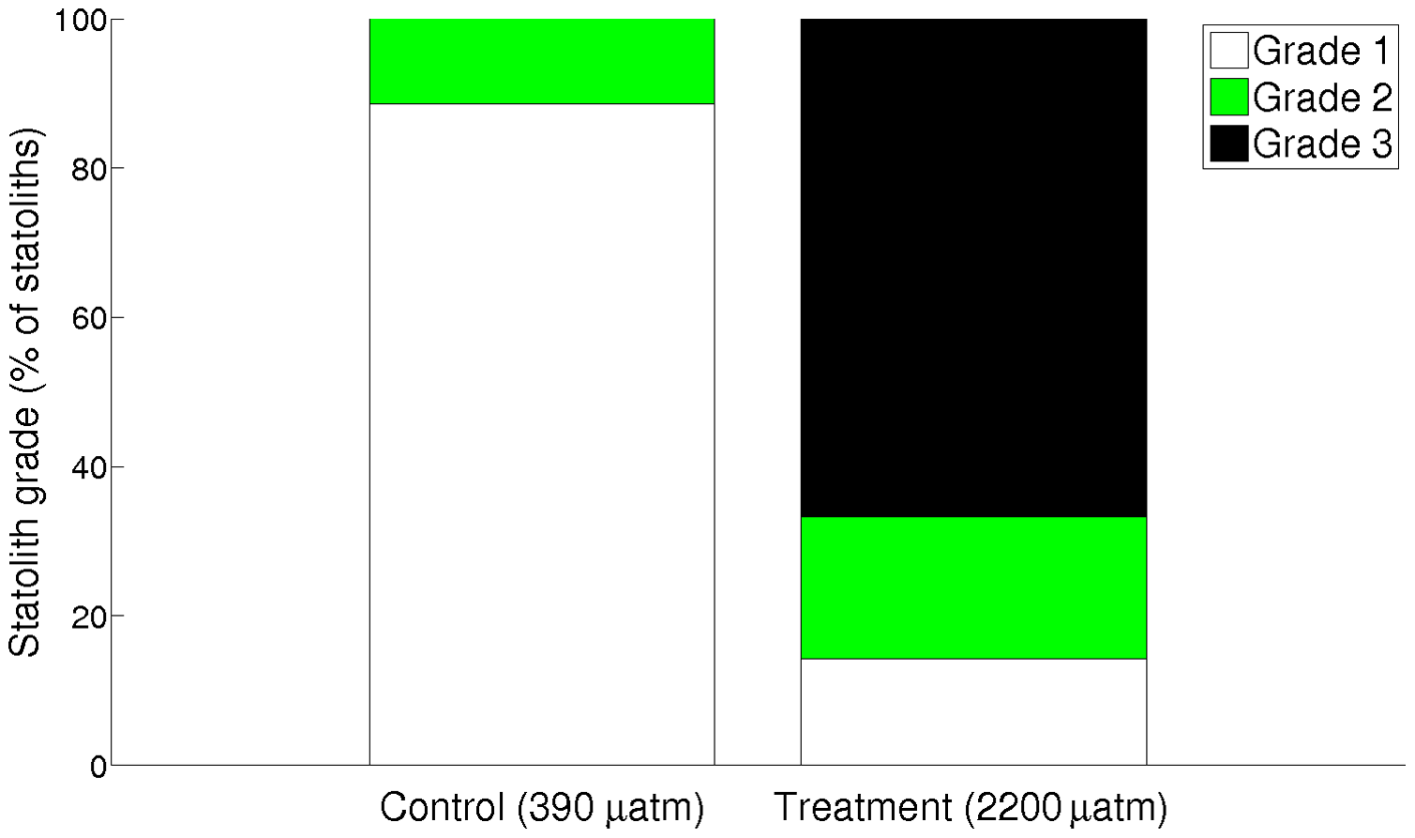
Statolith grades in two treatments. Statoliths were graded based on the following criteria outlined in Figure 1: (1) standard statolith shape and normal/minimal porosity, (2) standard shape with some abnormalities in the surface structure, slightly porous, and (3) porous and/or abnormal shape. The distribution of grades differed significantly between treatments (χ^2^ = 35.796, df  = 2, p<0.001), and statoliths removed from animals reared in treatment *p*CO_2_ were significantly more likely to be graded 2 or 3 and showed strong signs of abnormality and porosity.

Aragonite statoliths dissected from paralarvae reared in treatment *p*CO_2_ had 25% lower surface area (F_1,55_ = 70.722, p<0.001; Fig. 4). There was no significant difference between trials (F_1,54_ = 2.175, p<0.146). While statoliths from the control CO_2_ treatment were normally shaped, statoliths from high CO_2_ paralarvae typically exhibited abnormal and irregular shapes and were more porous (Fig. 5). In addition, SEM images of statoliths from the control treatment showed crystal size (submicron), shape (acicular), orientation (aligned along long-axes) and arrangement of crystal bundles (arrays radiating out from the primordium) that were typical of statoliths formed under ambient *p*CO_2_ conditions and consistent with paralarval statoliths of other squid species [48]. This highly organized ultra-structure was not observed in statoliths accreted under elevated *p*CO_2_. The distribution of grades given to statoliths varied significantly between treatments (χ^2^ = 35.796, df  = 2, p<0.001; Fig. 6).

## Discussion

We observed clear differences between *D. pealeii* paralarvae reared under control and treatment CO_2_ concentrations. Effects were found across all variables tested including hatching time, overall body size and internal aragonite statoliths. The spectrum of impacts suggests that young squid may be impacted by ocean acidification conditions projected for the end of this century [7]. Although the CO_2_ and pH levels in this study were high compared to near-term open ocean estimates [6] they fall within the range of future ocean predictions [1], [70], and of current near-shore variation [71]–[73]. While observations in this area are limited, data from two studies in different years and in different seasons suggests that the aragonite saturation state throughout the coastal range of *D. pealeii* can vary between 1.4–2.4, which is similar to values calculated for the control treatment (AOML-NEFSC collaborative project. Data collected on R/V Delaware II, analyzed and hosted by AOML, assembled by N. Rebuck; [74]), and pH may vary from 7.85–8.05 in Massachusetts shelf waters [74]. The species tested is a coastal squid that lives in relatively shallow waters during summer breeding seasons, which last from May to August in New England [47], [75]. Thus this experiment took place during the middle of the annual breeding season.

Under elevated *p*CO_2_ conditions, paralarval hatching was significantly delayed. Delayed hatching could result from metabolic suppression due to elevated *p*CO_2_ [76]. Cephalopod embryos develop in the perivitelline fluid (PVF) in which, due to gas diffusion properties of the eggshell, *p*CO_2_ is very high and *p*O_2_ very low [19]. Thus, the PVF may be undersaturated with respect to aragonite even under ambient pH conditions; under seawater *p*CO_2_ of ∼1400 μatm the PVF *p*CO_2_ could be as high as ∼4000 μatm [19]. As such, ocean acidification conditions may significantly increase the PVF pCO_2_, which may lead to additional metabolic costs (e.g. [14]). Delayed development in cuttlefish has been found when reared at ∼3700 μatm [41], and elevated seawater *p*CO_2_ has also been linked to longer development times in other marine invertebrate species [77], [78]. However, other rearing experiments on cephalopods have found no evidence for delayed development under seawater *p*CO_2_ ranging from 900 to 1400 μatm [42], suggesting that there may be threshold levels at which ocean acidification will affect cephalopods. Because squid egg sacs are a food resource for many fishes [28], extended development time would increase the probability of being preyed upon. In addition, increased development time may also put additional metabolic obligations on marine invertebrates that could lead to the depletion of egg yolk nutrients, with negative implications for survival.

Mantle length was significantly reduced in elevated *p*CO_2_-reared paralarvae but the differences were small. Similar results have been reported for cuttlefish reared at ∼3700 μatm [41]. In fish, reduced larval size at hatching increases the likelihood of predation due to extended development durations, and results in higher overall mortality rates [79], [80]. Fish larval swim speeds and distances travelled are positively correlated with larval size [81]. As squid paralarvae are highly mobile and migratory, reduced size may impact travel speed, ability to swim in currents, and migration distance. The relative differences in mantle length were more pronounced in trial 1 than trial 2. This heterogeneity may have resulted from a number of factors including pre-fertilization environmental and genetic differences between groups. Environmental conditions of the egg can impact larval size and development in fish and cephalopods [39], [82], and maternal condition may also affect egg quality [83]–[85]. Given that this experiment used eggs from female squid that were caught on two separate occasions, it is possible that the two groups had been exposed to different feeding opportunities, but prior to egg laying the captured squid were all fed the same fish diet and thus there were no dietary differences in the days immediately prior to spawning in the laboratory. Therefore, while female condition was uncertain, we do not expect that it was a major determinant in the impacts seen here. No differences in mantle length were found in *Loligo vulgaris* paralarvae when reared under 380, 850 and 1500 μatm *p*CO_2_ [42]. Thus, adverse effects of ocean acidification on cephalopods may only be present at significantly elevated *p*CO_2_ (i.e., the levels used in the present experiment and in that of Hu *et al*. [41]).

The control water was supersaturated with respect to aragonite (Ω_arag_≈1.7) and the treatment water was under-saturated (Ω_arag_≈0.5). In under-saturated conditions, aragonite should tend towards dissolution in seawater rather than precipitate out of it. Although several studies have shown that corals, molluscs and calcifying plants can continue to accrete aragonite structures even in under-saturated conditions (e.g. [15], [20], [86], [87]), undersaturation usually has strong, negative impacts on both the amount of aragonite these organisms produce and the morphology and organization of crystals (e.g. [20]). In this study, paralarval squid statoliths had significantly reduced surface area in animals reared under elevated *p*CO_2_. Conversely, in young fish and cuttlefish, both of which accrete internal aragonite structures, structure size and calcification rate have been shown to increase in low pH conditions [17], [19]. Calcification rates of the cuttlebone have been shown to increase in the cuttlefish at *p*CO_2_ ranging from ∼800 to ∼6000 μatm [18], [19] while cuttlebone density did not differ between *p*CO_2_ levels. Similar results have been shown for the squid *L. vulgaris*, in which statolith surface area increased under elevated *p*CO_2_ (∼850 and ∼1500 μatm) [42]. Unlike the variation in development times and mantle lengths between experiments, differences in the growth of calcified structures do not follow a clear trend across studies in terms of effects that vary with *p*CO_2_ level or life stage. As such, further investigation is necessary to better characterize the variability in cephalopod calcification across *p*CO_2_ levels, structures, developmental stage, and species.

In addition to reduced surface area, many statoliths removed from high *p*CO_2_-reared individuals in this experiment were malformed, and showed abnormal crystal structure. Squid statoliths, including those of *Doryteuthis* spp, typically consist of crystal bundles arranged in arrays radiating out from the primordium [88]–[91]. Here, the statolith crystal structure of control *p*CO_2_-reared hatchlings was consistent in size, shape and orientation with paralarval statoliths of other squids reared under control conditions [48]. However, high *p*CO_2_-reared paralarval statoliths lacked a highly organized ultra-structure, were more porous, and deviated from the typical droplet shape. Mis-shapen statoliths may have severe consequences for squid because the statolith is required for proper swimming and orientation as they migrate, find food and avoid predators. We were unable to obtain adequate videos of swimming behavior in this study, but malformed statoliths have been shown to disable swimming abilities [46], [92], and sensory abilities are impaired when statoliths are not present [93].

Our results suggest that exposure of embryos and early larval stages to elevated *p*CO_2_ and/or lowered pH may be particularly detrimental. However, this and other squid species dynamically migrate [47], [94] and encounter oceanographic regions of high CO_2_ during their lifetimes [76], [95]. Pacific Humboldt squid (*Dosidicus gigas*) are able to suppress their metabolism when vertically migrating through oxygen minimum zones [96]. Other squid, such as *Vampyroteuthis infernalis*, exist almost solely in these environments and thus are presumed to have vital rates adapted to higher CO_2_ and lower O_2_ levels [97]. The high reproductive output of most cephalopod species suggests that their potential for adaptation to a changing environment may be high. Results thus far suggest that there may be *p*CO_2_ threshold levels at which effects on cephalopod embryonic development time and size-at-hatching are demonstrated [41]. Indeed, some studies that have examined ocean acidification effects on older (i.e., juvenile) animals or that have used lower *p*CO_2_ levels have found no such effects [18], [42], [43]. The cephalopod calcification response to ocean acidification does not appear to follow a clear trend, which suggests that further investigations with other squid species under similar experimental conditions are required to determine the range of responses. Likewise, experiments across a range of CO_2_ concentrations will be needed in order to determine whether there are threshold ocean acidification levels for impacts on calcification rates in cephalopods.
